# Antenatal and postpartum depression among women who conceived after pregnancy loss: a longitudinal study

**DOI:** 10.1007/s00737-025-01632-8

**Published:** 2025-10-09

**Authors:** Yuka Ito, Natsu Sasaki, Kazuhide Tezuka, Kotaro Imamura, Daisuke Nishi

**Affiliations:** 1 Department of Mental Health, Graduate School of Medicine, The University of Tokyo, 7-3- 1, Hongo, Bunkyo-ku, Tokyo, Japan; 2 Department of Digital Mental Health, Graduate School of Medicine, The University of Tokyo, 7-3-1, Hongo, Bunkyo-ku, Tokyo, Japan

**Keywords:** Pregnancy loss, Antenatal depression, Postpartum depression, Mental health, Childbirth

## Abstract

**Purpose:**

Although the relationship between previous pregnancy loss and perinatal depression has been explored, most previous research has been cross-sectional and has not utilized diagnostic evaluation techniques. This study longitudinally examined the relationship using the self-administered web-based World Health Organization Composite International Diagnostic Interview 3.0.

**Methods:**

This study used data from a control group of pregnant women in a randomized controlled trial conducted between November 2019 and March 2020. An accelerated failure time model with Weibull distribution was conducted to evaluate the impact of previous pregnancy loss by number (never, once, and two or more times) on the onset of perinatal depression from 18 ± 2 weeks (baseline) to 3 months postpartum.

**Results:**

The final analysis included 2,347 participants. The risk of developing perinatal depression was significantly higher for those with two or more previous pregnancy losses compared to those with no previous pregnancy loss (adjusted models: time ratio 0.17, 95% CI 0.03–0.86, *p* = 0.033). No statistically significant difference was found between those with one pregnancy loss and those with no previous pregnancy loss (adjusted models: time ratio 0.99, 95% confidence interval [CI] 0.24–4.04, *p* = 0.990).

**Conclusion:**

Women who experienced repeated pregnancy loss had an elevated risk of diagnosable perinatal depression. Thus, it is crucial to consider interventions targeting pregnant women who have experienced repeated pregnancy loss to prevent perinatal depression.

## Highlights

- A single previous pregnancy loss did not elevate the risk of diagnosable perinatal depression in subsequent pregnancies.
- Repeated pregnancy loss is associated with an increased risk of diagnosable perinatal depression.
- Healthcare providers should assess pregnancy loss history and provide appropriate care in subsequent pregnancies.

## Introduction

The perinatal phase, which involves the transition to motherhood, is a critical period characterized by significant biological and psychological changes among women. These changes increase susceptibility to mental disorders, including depression (Callaghan et al. 2024; Dagher et al. 2021; Mercer 2004). Perinatal depression is a crucial public health concern that has serious implications for both mothers and children, and is usually associated with premature birth, maternal suicide, child maltreatment, and delayed child development (Brockington 2017; Chaffin et al. 1996; Grigoriadis et al. 2013; Grote et al. 2010; Rogers et al. 2020; Stein et al. 2014). Therefore, it is crucial for health care practitioners to recognize the potential risk factors for the early detection and treatment of perinatal depression. For example, relatively well-established risk factors for perinatal depression include low education, low income, unintended pregnancy, poor social support, stressful life events, and a history of depression (Leigh and Milgrom 2008; Norhayati et al. 2015; O’hara and McCabe 2013; Sexton et al. 2012; Yim et al. 2015). Thus, additional risk factors must be investigated and applied in clinical practice.

Pregnancy loss, including miscarriage and induced abortion, is perceived as a difficult event in life (Bardos et al. 2015; Kersting and Wagner 2012). It affects approximately 15–25% of women of reproductive age (Genovese and McQueen 2023; Price et al. 2006; Quenby et al. 2021), highlighting the necessity for greater recognition and support. Women who undergo pregnancy loss are more prone to suffer from mental health issues like depression and anxiety (Bellieni and Buonocore 2013; Fergusson et al. 2008; Herbert et al. 2022; Lok et al. 2010). Therefore, mental support has been advocated for women experiencing pregnancy loss, whether induced or involuntary (Kong et al. 2010; Lancet 2008). As the rate of subsequent pregnancies and childbearing among women who have experienced pregnancy loss is high (Greenberg et al. 2015; Stubblefield et al. 1984; Wong et al. 2015), research on whether previous pregnancy loss affects the development of perinatal depression is essential. Previous studies have reported that pregnancy loss is linked to an increased risk of experiencing depressive symptoms during subsequent pregnancies and postpartum periods (Blackmore et al. 2011; Bradley 1984; Chojenta et al. 2014; Giannandrea et al. 2013; Gong et al. 2013; Kumar and Robson 1978). These studies have included reports that repeated pregnancy loss is associated with a greater risk of perinatal depression (Blackmore et al. 2011; Giannandrea et al. 2013).

However, almost all existing research investigating the relationship between past pregnancy loss and　perinatal depression uses screening tools for depressive symptoms and considers depression when the cutoff thresholds are exceeded. Although this is a commonly used method, false positives are common, the prevalence is exaggerated (Levis et al. 2019; Thombs et al. 2018), and there is a strong need for studies using diagnostic assessment tools. Only Giannandrea et al. (2013) explored the association between pregnancy loss and perinatal depression using diagnostic assessment tools. However, they assessed depression among low-income pregnant women only, within the first year postpartum and without limiting the period, which has methodological limitations. Furthermore, many women experience repeated pregnancy losses, and previous studies have shown that pregnant women with two or more previous pregnancy losses do not experience lower anxiety levels throughout their pregnancies, unlike those with only one previous loss (Fertl et al. 2009). Therefore, it is crucial to determine the impact of two or more pregnancy losses on perinatal depression. Nevertheless, to our knowledge, no studies have examined the impact of the frequency of pregnancy loss on perinatal depression throughout pregnancy and the postpartum period using diagnostic assessment tools.

Therefore, the aim of this study was to longitudinally explore the impact of previous pregnancy losses by frequency on depression throughout subsequent pregnancies and the postpartum period, using the self-administered version of the World Health Organization Composite International Diagnostic Interview 3.0 (WHO-CIDI 3.0) among a diverse group of women.

## Materials and methods

### Study design and participants

Data for this longitudinal study were derived from the control group of a randomized controlled trial (RCT) of a 12-week cognitive behavioral therapy program aimed at preventing perinatal depression in pregnant women (Nishi et al. 2022). To eliminate the impact of physical symptoms during early pregnancy on mental health and to assess the effectiveness of the intervention in late pregnancy, the eligibility criteria were set at 16–20 weeks of gestation. The research protocol was previously published (Nishi et al. 2020) and authorized by the Research Ethics Review Board at the Graduate School of Medicine and Faculty of Medicine, University of Tokyo (No. 2019150NI). The study was issued with the identifier UMIN000038190 in the Clinical Trials Registry maintained by the University Hospital Medical Information Network (UMIN). Participants were recruited via a pregnancy tracking application named “Luna Luna Baby” developed by MTI Ltd. The criteria for included participants were as follows: (a) female; (b) over 20 years old; (c) at 18 ± 2 weeks gestation; (d) not meet the diagnostic criteria for a major depressive episode (MDE) within the preceding month, based on the web-based WHO-CIDI 3.0; and (e) not diagnosed with bipolar disorder. All participants provided informed consent through an internet survey, and their anonymity was preserved. The reporting of this study complied with the Strengthening the Reporting of Observational Studies in Epidemiology (STROBE) statement (Vandenbrouckel, et al., 2007).

**Fig. 1:**
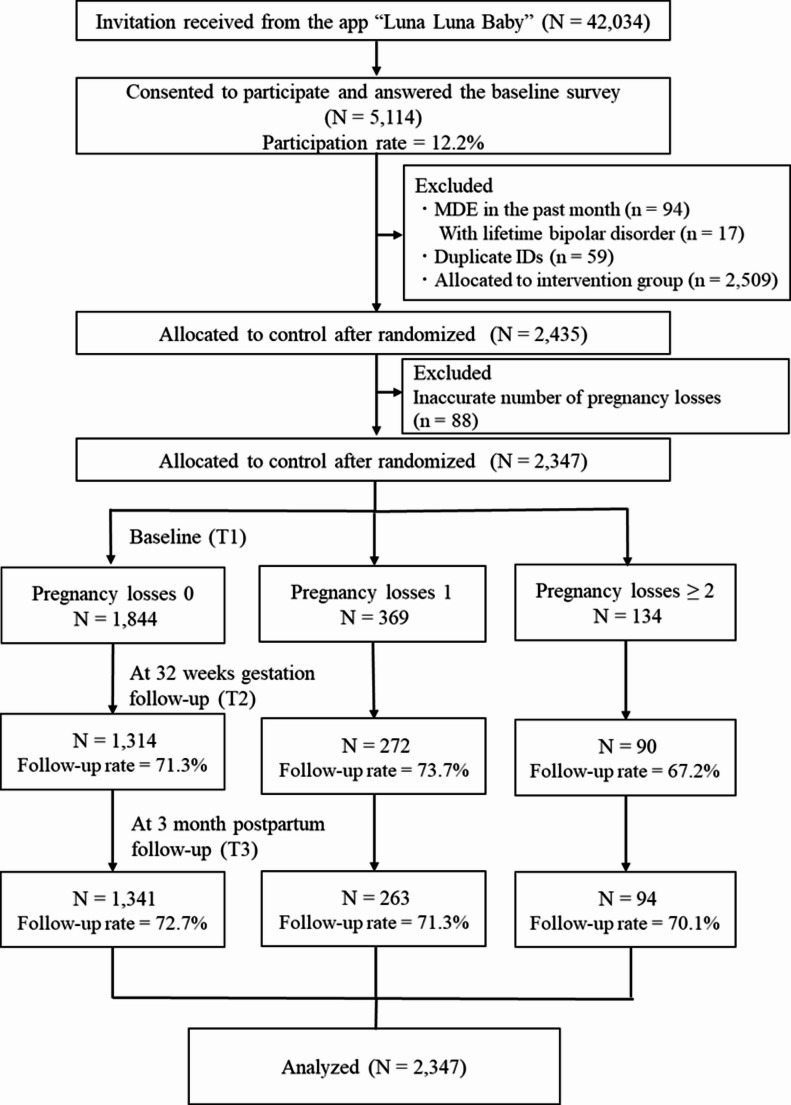
Participant recruitment flowchart

Figure 1 illustrates a flowchart of the participants. A total of 2,435 women were allocated to the control arm of the RCT, of which 88 were excluded owing to an inaccurate number of pregnancy losses. Thus, the analytical sample comprised a total of 2,347. The numbers of previous pregnancy losses were 1,844, 369, and 134 for none, one, and two or more losses, respectively.

### Measurements

#### Pregnancy loss

In this study, we used the number of pregnancy losses before 22 weeks, based on the WHO definition of miscarriage and the period during which abortion is legally available in Japan. The number was determined by subtracting the number of previous births after 22 weeks from the number of previous pregnancies. This is because the number of miscarriages and abortions was not directly measured in this study. It is important to note that this approach combines both induced and spontaneous losses, which could introduce heterogeneity (Bellieni and Buonocore 2013; Fergusson et al. 2008).

#### Incidence of MDEs

The primary outcome measure was the occurrence of MDEs by 32 weeks of gestation and by three months postpartum. The incidence of MDEs throughout the follow-up period was assessed utilizing the self-administered web-based WHO-CIDI 3.0 depression section, which is based on the DSM-IV-TR criteria. The web-based version showed good agreement with the clinical diagnosis of MDEs (Peters et al. 1998). MDE onset was defined as a participant reporting an episode of an MDE at T2 (32 weeks gestation) or T3 (three months postpartum) after the baseline (18 ± 2 weeks gestation). Information on the month of MDE onset was also collected.

#### Covariates

Several covariates were included in our model: age, education (high school or lower/university or higher), employment (unemployed/student/leave of absence/part-time/full-time), partner (yes/no), history of mental illness (yes/no), number of children, and history of infertility treatment (yes/no).

### Statistical analysis

To describe the demographic features of the cohort, mean scores and standard deviations were employed for continuous variables, while frequencies and proportions were used for categorical variables. We performed an accelerated failure time (AFT) model with Weibull distribution to evaluate the impact of previous pregnancy loss on the onset of perinatal depression (Wei 1992). Considering the potential time-dependent effects of pregnancy loss experience, we selected this model which does not require the proportional hazards assumption. The AFT model directly models time to event and provides time ratios indicating how predictors affect the time to depression onset. Time ratios and 95% confidence intervals (CIs) for MDE incidence according to the number of pregnancy losses experienced were estimated in both crude and adjusted models, controlling for the above covariates. Each participant’s follow-up time was expressed as either the number of months from T1 (baseline) to MDE onset or the end of the follow-up period (T3 or T2 if the participant dropped out at the T3 follow-up), whichever occurred first. Participants who did not develop MDE by the end of the follow-up period were considered as censored cases. Those who were lost to follow-up at T3 were also treated as censored cases, with their follow-up time calculated from T1 to T2. R (version 4.5.1; R Core Team, Vienna, Austria) was employed to analyze the data.

### Results

The demographic characteristics of the participants are presented in Table 1. Participants’ average age was 30.5 years. Roughly half of the participants graduated from university or graduate school and were full-time workers. Participants with pregnancy loss were older, had fewer college degrees or higher, tended to not work, and were more likely to be multiparous. A higher proportion of the group with a single pregnancy loss had a history of mental illness. Participants in the group with two or more pregnancy losses were more likely to have no partner and to have become pregnant through fertility treatment. In total, 49 (2.66%) participants in the no pregnancy loss group, 11 (2.98%) in the one pregnancy loss group, and 9 (6.72%) in the two or more pregnancy loss group reported a new MDE during the follow-up period.

**Table 1:** Demographic characteristics by pregnancy loss frequency

Baseline Characteristic	Number of previous pregnancy losses		
	0 (*N* = 1,844, 78.6%)	1 (*N* = 369, 15.7%)	≥ 2 (*N* = 134, 5.7%)
	*n* (%)		
Number of previous pregnancy losses			
0	1844(100)		
1		369(100)	
2			105(78.4)
3			19(14.2)
4			8(6.0)
5			2(1.5)
Age, Mean (SD)	30.1(4.45)	30.3(4.53)	33.6(4.49)
20–29 years old	868(47.1)	138(37.4)	30(22.4)
30–39 years old	936(50.8)	214(58.0)	91(67.9)
40–49 years old	40(2.2)	17(4.6)	13(9.7)
Education status			
Junior high school	38(2.1)	19(5.1)	7(5.2)
High school	609(33)	147(39.8)	66(49.3)
College	229(12.4)	32(8.7)	17(12.7)
University	888(48.2)	152(41.2)	40(29.9)
Graduate school	80(4.3)	19(5.1)	4(3.0)
Employment status			
Unemployed	377(20.4)	99(26.8)	39(29.1)
Student	15(0.8)	1(0.3)	0(0.0)
Leave of absence	106(5.7)	26(7)	6(4.5)
Part-time	281(15.2)	58(15.7)	24(17.9)
Full-time	1065(57.8)	185(50.1)	65(48.5)
Partner			
With partner	1834(99.5)	365(98.9)	130(97.0)
Without partner	10(0.5)	4(1.1)	4(3.0)
History of mental illness			
No	1614(87.5)	302(81.8)	112(83.6)
Yes	230(12.5)	67(18.2)	22(16.4)
Number of children			
0	1294(70.2)	214(58.0)	62(46.3)
1	411(22.3)	106(28.7)	43(32.1)
2	119(6.5)	37(10)	19(14.2)
3	19(1.0)	10(2.7)	8(6.0)
4 or more	1(0.1)	2(0.5)	2(1.4)
History of infertility treatment			
No	1569(85.1)	305(82.7)	103(76.9)
Yes	275(14.9)	64(17.3)	31(23.1)

The results of the AFT model analysis are presented in Table 2. In the crude model, women with two or more pregnancy losses had significantly shorter time to MDE onset compared to those without pregnancy loss (time ratio 0.13, 95% confidence interval [CI] 0.03–0.62, *p* = 0.011). After adjusting for covariates, this association remained significant (time ratio 0.17, 95% CI 0.03–0.86, *p* = 0.033).

**Table 2:** Association between major depressive episode and variables (Accelerated failure time model with Weibull distribution)

	Crude		Adjusted	
	Time ratio (95% CI)	***p***	Time ratio (95% CI)	***p*** ^a^
Number of previous pregnancy loss				
0	ref.		ref.	
1	0.80 (0.20–3.22)	0.755	0.99 [0.24–4.04]	0.990
≥ 2	0.13 (0.03–0.62)	0.011*	0.17 [0.03–0.86]	0.033*
Age			1.01 [0.89–1.14]	0.854
Education status				
high school or lower			ref.	
university or higher			0.99 [0.35–2.84]	0.990
Employment status				
Unemployed			ref.	
Student			0.26 [0.00-19.86]	0.539
Leave of absence			1.81 [0.18–18.24]	0.617
Part-time			1.35 [0.27–6.70]	0.714
Full-time			1.66 [0.48–5.77]	0.423
Partner				
With partner			ref.	
Without partner			1.97 [0.03-135.29]	0.753
History of mental illness				
No			ref.	
Yes			0.18 [0.05–0.62]	0.007*
Number of children			0.84 [0.39–1.78]	0.639
History of infertility treatment				
No			ref.	
Yes			0.41 [0.11–1.55]	0.190

^a^ Adjusted for age, education, employment, partner, history of mental illness, number of children and history of infertility treatment

CI: confidence interval

* p < 0.05

There was no statistically significant difference in the time to MDE onset between those with one pregnancy loss and those without a history of pregnancy loss (adjusted: time ratio 0.99, 95% CI 0.24–4.04, *p* = 0.990). In addition to experiencing pregnancy loss, participants with a history of mental illness had significantly shorter time to MDE onset (adjusted: time ratio 0.18, 95% CI 0.05–0.62, *p* = 0.007), indicating an increased risk of developing MDEs during the perinatal period.

## Discussion

As far as we know, this study is the first to longitudinally investigate the risk of developing MDEs from the gestational to the perinatal period after experiencing pregnancy loss using a reliable diagnostic assessment tool. A single previous pregnancy loss did not increase the risk of developing an MDE throughout the perinatal period. However, two or more previous pregnancy losses were associated with shorter time to MDE onset.

The results of this study differ from those of previous studies that have examined pregnancy loss and perinatal depression using screening tools. These studies reported that experiencing even one pregnancy loss significantly predicts perinatal depressive symptoms (Blackmore et al. 2011; Bradley 1984; Chojenta et al. 2014; Giannandrea et al. 2013; Gong et al. 2013; Kumar and Robson 1978). This could be due to the high number of false positives in the screening tool (Levis et al. 2019; Thombs et al. 2018). The only study that investigated the association between pregnancy loss and postpartum depression using diagnostic assessment tools, conducted by Giannandrea et al. (2013), demonstrated that a history of one pregnancy loss increased the risk of depression significantly in the first year after childbirth. However, that study was a cross-sectional study targeting pregnant women at high risk of depression who were in economically disadvantaged circumstances, and thus had limitations. This study expands on their findings and may be more generally applicable to pregnant women in general. Furthermore, current study suggests the need to pay sufficient attention to recurrent pregnancy loss, which may increase the risk of MDE at the diagnostic level.

Repeated pregnancy loss is a traumatic and profoundly distressing event in a woman’s life (Giannandrea et al. 2013; Lee and Slade 1996; Slobodin 2014), and several potential mechanisms can explain the increased risk of perinatal depression. First, women who have experienced recurrent miscarriage may experience higher levels of anxiety throughout pregnancy compared to women who have experienced a single miscarriage, due to doubts about the likelihood of a successful pregnancy (Fertl et al. 2009), which may contribute to the development of depression during pregnancy in particular. (Gao et al. 2020; Kessler et al. 1996; Starr and Davila 2012; Starr et al. 2014; Wittchen et al. 2000).

Another potential mechanism is grief. Pregnancy loss, particularly in the case of miscarriage, is characterized as an unexpected and sudden event. Additionally, individuals who have experienced pregnancy loss tend to receive less psychosocial support compared to those who have experienced other types of grief (Simmons et al. 2006). Therefore, despite intense grief resulting from loss, the grief process may not progress (Davoudian et al. 2021). Previous reports linking the number of miscarriages to the intensity of grief (Johnson and Johnston et al.^2021^) responses may help explain the current findings, and further research is needed.

Third, some women, particularly those who have experienced abortion, may have a reactivation of previously suppressed grief in a subsequent pregnancy. While this concept of “reactivated grief” reported by Kumar and Robson (1978) has not received significant attention recent years (Casey 2010), it may play a role in the development of perinatal depression. Moreover, previous research has noted an association between abortion and adverse childhood experiences (Kanamori et al. 2023; Steinberg and Tschann 2013). Pregnancy loss may be an additional traumatic experience and the overall burden of trauma may increase the levels of perinatal depression.

A secondary finding of this study is that a history of psychiatric illness is also a predictor of perinatal depression. This is consistent with the strong evidence from earlier research (Guintivano et al. 2018; Johansen et al. 2020; Suri et al. 2017).

This study has important implications in that a history of repeated pregnancy loss should be considered a risk factor for perinatal depression. It is crucial for healthcare providers to assess pregnancy loss history in their daily clinical practice, which can be performed with less reporting bias and in a short time. Qu et al. (2021) showed that social support buffers depression during subsequent pregnancies among women who have experienced repeated pregnancy losses. Donegan et al. (2023) stated that pregnant women who conceived after perinatal loss highly valued compassionate care delivered by healthcare providers. Conversely, insensitive and neglectful attitudes can have a negative impact (Cullen et al. 2017). Therefore, as noted by Donegan et al. (2023), training healthcare providers to care for women at the time of loss and during subsequent pregnancies is important.

### Limitations

This study has several limitations. First, the application users are not representative of all pregnant women, thereby limiting generalizability of the findings. Second, participants were asked retrospectively about the number of previous pregnancies and deliveries, which may have introduced recall bias. However, the objective character of the event offers some defense against this possibility. To mitigate this bias in future studies, researchers could consider validating self-reported data with medical records. Third, the number of MDE cases in each group was small, and the results may not be robust. However, the sample size was larger than that reported in a previous study that used diagnostic assessment tools. Future studies with larger sample sizes are needed to confirm our findings. Fourth, several potential confounding variables, such as income, adverse childhood experiences, mode of delivery, and partner social support, were not included in the model because these variables were not measured in the current study. Future studies should aim to include these important covariates to provide a more comprehensive understanding of the factors contributing to perinatal depression. Fifth, this study did not allow for comparisons by type of individual pregnancy loss (e.g., induced abortion or miscarriage). In clinical practice, the details of past pregnancy losses are often difficult to obtain rigorously because of their sensitivity. Therefore, it is an important finding that the present study suggests that repeated pregnancy loss may influence subsequent perinatal depression, regardless of the type of pregnancy loss. However, there may be differences in the risk of depression onset, mechanisms of onset, and management strategies between abortion and miscarriage (Bellieni and Buonocore 2013), necessitating further research in the future. Finally, the effect of the time since loss as a possible predictor of postpartum mood could not be evaluated. Nonetheless, this study demonstrated the impact of repeated pregnancy loss regardless of the time of pregnancy.

## Conclusion

Women who experienced previous repeated pregnancy loss had an elevated risk of diagnosable depressive disorder during the perinatal period compared with those who did not experience pregnancy loss. It is important to explore the specific psychological effects of different types of pregnancy loss and to develop and evaluate the effectiveness of interventions tailored to their needs in future research.

## Data Availability

The data that support the findings of this study are available from the corresponding author, DN, upon reasonable request.

## Declarations

### Clinical trial registration

N/A.
